# The 10-Repeat 3′-UTR VNTR Polymorphism in the *SLC6A3* Gene May Confer Protection Against Parkinson’s Disease: A Meta-analysis

**DOI:** 10.3389/fgene.2021.757601

**Published:** 2021-09-27

**Authors:** Qiaoli Zeng, Fan Ning, Shanshan Gu, Qiaodi Zeng, Riling Chen, Liuquan Peng, Dehua Zou, Guoda Ma, Yajun Wang

**Affiliations:** ^1^ Maternal and Children’s Health Research Institute, Shunde Women and Children’s Hospital, Guangdong Medical University, Foshan, China; ^2^ Key Laboratory of Research in Maternal and Child Medicine and Birth Defects, Guangdong Medical University, Foshan, China; ^3^ Institute of Neurology, Affiliated Hospital of Guangdong Medical University, Zhanjiang, China; ^4^ Department of Clinical Laboratory, People’s Hospital of Haiyuan County, Zhongwei, China; ^5^ Department of Pediatrics, Shunde Women and Children’s Hospital, Guangdong Medical University, Foshan, China; ^6^ Institute of Respiratory, Shunde Women and Children’s Hospital, Guangdong Medical University, Foshan, China

**Keywords:** Parkinson’s disease, Slc6a3, dopamine transporter, variable number of tandem repeats, meta-analysis

## Abstract

The dopamine transporter (DAT) is encoded by the SLC6A3 gene and plays an important role in the regulation of the neurotransmitter dopamine. The SLC6A3 gene contains several repetition alleles (3–11 repeats) of a 40-base pair variable number of tandem repeats (VNTR) in the 3′-untranslated region (3′-UTR), which may affect DAT expression levels. The 10-repeat (10R) allele could play a protective role against PD. However, inconsistent findings have been reported.

**Methods:** A comprehensive meta-analysis was performed to accurately estimate the association between the 10R allele of the 3′-UTR VNTR in SLC6A3 and PD among four different genetic models.

**Results:** This meta-analysis included a total of 3,142 patients and 3,496 controls. We observed a significant difference between patients and controls for the allele model (10R vs. all others: OR = 0.860, 95% CI: 0.771–0.958, P = 0.006), pseudodominant model (10R/10R + 10R/9R vs. all others: OR = 0.781, 95% CI: 0.641–0.952, P = 0.014) and pseudorecessive model (10R/10R vs. all others: OR = 0.858, 95% CI: 0.760–0.969, P = 0.013) using a fixed effects model. No significant differences were observed under the pseudocodominant model (10R/9R vs. all others: OR = 1.079, 95% CI: 0.945–1.233, P = 0.262). By subgroup analysis, the 10R, 10R/10R and 10R/9R genotypes were found to be significantly different from PD in Asian populations.

**Conclusion:** Our findings suggest that the *SLC6A3* 10R may be a protective factor in susceptibility to PD.

## Introduction

Parkinson’s disease (PD) is a very common neurodegenerative disorder. One of the critical neuropathologies of PD is the degeneration of dopamine-producing neurons in the substantia nigra, resulting in impairment of the dopaminergic pathway and the subsequent depletion of dopamine levels (Balestrino and Schapira 2020). Another pathologic hallmark is the presence of ubiquitinated protein deposits named Lewy bodies, which cause dopaminergic cell death (Balestrino and Schapira, 2020; Singleton et al., 2003). These pathologic changes result in depletion of dopamine levels that underlie the etiology of PD. Therefore, dopaminergic transmission and metabolism pathway genes have been investigated and are considered to be candidate genes for PD.

The dopamine transporter (DAT) plays an important role in dopaminergic neurotransmission. It is mainly present on the terminals of neurons in the substantia nigra and is responsible for controlling the duration and intensity of neurotransmission by rapid dopamine uptake into the presynaptic terminals; thus, DAT is critical in the temporal and spatial buffering of released dopamine and its recycling (Cheng and Bahar 2015; Uhl 2003; Bannon et al., 2001). In addition, DAT is considered a gateway for neurotoxicants because the nigrostriatal toxicant 1-methyl-4-phenylpyridinium (Mpp^+^) is taken up selectively by presynaptic DAT, and access to dopaminergic neurons leads to dopaminergic cell toxicity (Krontiris 1995; Uhl et al., 1994); DAT has also been shown to interact with alpha-synuclein (a kind of Lewy body) (Lee et al., 2001; Wersinger et al., 2003; Thomas and Beal 2007). These findings provide evidence for a role of DAT in PD and seem to explain why the density of DAT correlates with the extent of dopaminergic cell loss in PD brains.

DAT is coded by the *SLC6A3* gene. The 3′-UTR of the *SLC6A3* gene includes a 40 bp variable number tandem repeat (VNTR) polymorphism. Between 3 and 11 copies of the 40 bp VNTR have been identified in normal populations (Uhl 2003), and the 9 and 10 repeat alleles are most frequent in both PD patients and several populations (Uhl 2003; Bannon et al., 2001). The *SLC6A3* VNTR itself seems to be a functional polymorphism. A recent study found that the seed region of miR-491 is located in the VNTR fragment of the DAT mRNA e 3′-UTR, and the effect of miR-491 on DAT expression is dependent on the VNTR copynumber (Jia et al., 2016). Thus, *SLC6A3* polymorphic VNTRs may directly influence DAT expression (Fuke et al., 2001; Heinz et al., 2002; Mill et al., 2002; Lynch et al., 2003). Lin reported that the 10R alleles conferred protection against PD compared to other alleles (Lin et al., 2003), but other noteworthy studies showed different results. Given these controversial conclusions, we performed a meta-analysis to systematically, quantitatively, and objectively summarize the association between the 10R of the 3′-UTR VNTR in *SLC6A3* and PD susceptibility.

## Materials and Methods

### Literature Search

The PubMed, Google Scholar, and Chinese National Knowledge Infrastructure databases were systematically searched for potentially qualified studies using a combination of the keywords “dopamine transporter,” “DAT,” “DAT1,” “*SLC6A3*,” “VNTR,” “3′-UTR,” “polymorphism,” “rs28363170” and “Parkinson.” with no language or date restrictions. All studies were evaluated on the basis of the title and abstract and we excluded studies that were clearly irrelevant. Then, potentially eligible studies were reviewed in full to determine the inclusion in the meta-analysis.

### Inclusion and Exclusion Criteria

Studies included in the meta-analysis had to meet all the following inclusion criteria: 1) case-control or cohort studies that evaluated the associations between the *SLC6A3* 3′-UTR VNTR polymorphism and the risk of PD; 2) available data for estimating odds ratios (ORs) with corresponding 95% confidence intervals (CIs); and 3) studies in which all PD patients had been diagnosed according to the common diagnostic criteria (Jankovic 2008).

Studies were excluded from the current analysis with the following criteria: 1) not a case-control or cohort study; 2) irrelevant to PD or *SLC6A3* 3′-UTR VNTR; 3) genotype distribution of the control subjects is not in Hardy-Weinberg equilibrium (HWE); and 4) reports lacking detailed genotype data.

### Data Extraction

The following data were independently extracted from the included studies and entered into a database to ensure the validity of the data: first author’s name, year of publication, ethnicity, number of patients and controls, allele distribution, and genotype distribution. Studies were excluded if they did not provide the above information.

### Statistical Analysis

Four genetic models were used in the meta-analysis: the allele model (10R vs. all others), the pseudodominant model (10R/10R + 10R/9R vs. all others), the pseudorecessive model (10R/10R vs. all others), and the pseudocodominant model (10R/9R vs. all others). Genetic heterogeneity was evaluated using the Q-test and I^2^ test. I^2^ ranged from 0 to 100%. Significant heterogeneity was defined with *p* < 0.01 and I^2^ > 50% (He et al., 2015; Shen et al., 2015; Zhang et al., 2016). If there was no significant heterogeneity among the total of studies, ORs with corresponding 95% CIs were calculated by the fixed effect model (Mantel–Haenszel); otherwise, ORs were calculated by a random-effect model. Z test was used to determine the significance of OR. Additionally, publication bias was investigated with Egger’s test and Begg’s test (Li et al., 2016; Liu et al., 2017; Han et al., 2019). Statistical analyses were performed using STATA v.16.0 software (Stata Corporation, Texas, United States).

## Results

### Study Inclusion and Characteristics

**FIGURE 1:**
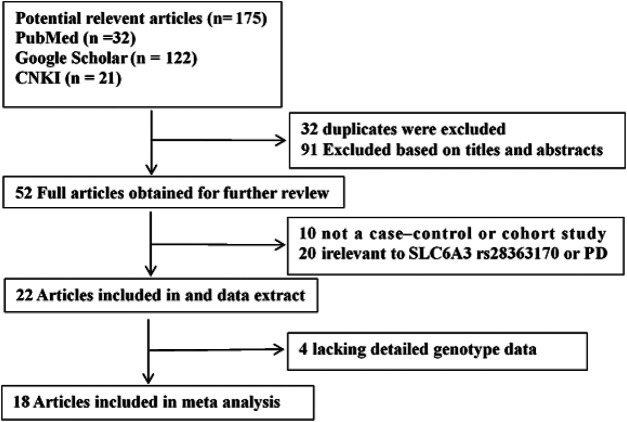
Flow diagram of the literature search and selection.

A total of 175 potential studies were retrieved through the initial search. Thirty-two duplicates were excluded. Then, 143 studies were screened on title and abstract, 91 of which were excluded. The remaining 52 articles were evaluated by full-text reading, 34 of which were excluded because 10 were not case-control or cohort studies, 20 were not related to the *SLC6A3* 3′-UTR VNTR or PD, and 4 did not provide sufficient data. A flow chart of study selection in the meta-analysis is shown in Figure 1. There were 18 potentially relevant papers, including 14 in English and 4 in Chinese; among them, 15 studies provided allele model data (Chang et al., 2018; Lu et al., 2016; Benitez et al., 2010; Kelada et al., 2005; Zhao et al., 2004; Lin et al., 2003; Goudreau et al., 2002; Kimura et al., 2001; Kim et al., 2000; Zhang et al., 2000; Wang et al., 2000; Mercier et al., 1999; Leighton et al., 1997; Le Couteur et al., 1997; plante-Bordeneuve et al., 1997), and 15 studies had pseudodominant, pseudorecessive and pseudoadditive model data (Chang et al., 2018; Lu et al., 2016; Benitez et al., 2010; Ritz et al., 2009; Kelada et al., 2005; Zhao et al., 2004; Lin et al., 2003; Lynch et al., 2003; Kim et al., 2000; Zhang et al., 2000; Wang et al., 2000; Mercier et al., 1999; Nicholl et al., 1999; Leighton et al., 1997; Le Couteur et al., 1997). The characteristics of each study are shown in Table 1.

**TABLE 1:** Characteristics of each study included in this meta-analysis.

Author	Year	Ethnic	Case/Control	Allele distribution						Genotype distribution							
				Cases, n			Control, n			Cases, n				Control, n			
				9R	10R	Other	9R	10R	Other	9R/9R	9R/10R	10R/10R	Other	9R/9R	9R/10R	10R/10R	Other
Chang et al.	2018	Chinese	52/60	19	85	0	23	97	0	5	9	38	0	6	11	43	0
Lu et al.	2016	Chinese	521/502	76	966	0	66	938	0	6	64	451	0	6	54	442	0
BENITEZ et al.	2010	South American	99/131	37	161	0	59	203	0	3	31	65	0	5	49	77	0
Ritz et al.	2009	Latino, Asian, and Native American	324/334	—	—	—	—	—	—	28	113	179	4	16	109	200	9
Kelada et al.	2005	non-Hispanic Caucasian	251/355	147	346	9	179	525	6	23	101	119	8	28	120	202	5
Zhao et al.	2004	Chinese	138/184	10	249	17	16	341	11	0	10	113	15	1	13	160	10
Lin et al.	2003	Chinese	193/254	32	342	12	30	465	13	1	29	151	12	1	26	214	13
Lynch et al.	2003	African-American, and Other	100/63	—	—	—	—	—	—	10	44	42	4	4	24	32	3
Goudreau et al.	2002	Caucasian	183/146	114	249	3	76	211	5	—	—	—	—	—	—	—	—
Kimura et al.	2001	Japanese	204/300	17	371	20	25	551	24	—	—	—	—	—	—	—	—
Kim et al.	2000	Korean	116/128	32	179	21	37	209	10	12	7	84	13	15	6	101	6
Zhang et al.	2000	Chinese	128/85	13	231	12	2	156	12	0	13	104	11	0	2	73	10
Wang et al.	2000	Chinese	171/180	20	300	22	13	333	14	0	20	130	21	0	13	153	14
Mercier et al.	1999	French	75/78	48	99	3	52	102	2	10	26	36	3	8	36	32	2
Nicholl et al.	1999	Caucasian	206/206	—	—	—	—	—	—	15	73	113	5	17	86	100	3
Leighton et al.	1997	Chinese	203/230	28	366	11	35	415	10	0	27	164	12	2	31	187	10
Le Couteur et al.	1997	Caucasian	100/200	51	144	5	112	286	2	7	36	52	5	15	81	102	2
Plante-Bordeneuve et al.	1997	British,French	78/60	42	108	6	34	85	1	—	—	—	—	—	—	—	—

### Heterogeneity Analysis

**FIGURE 2:**
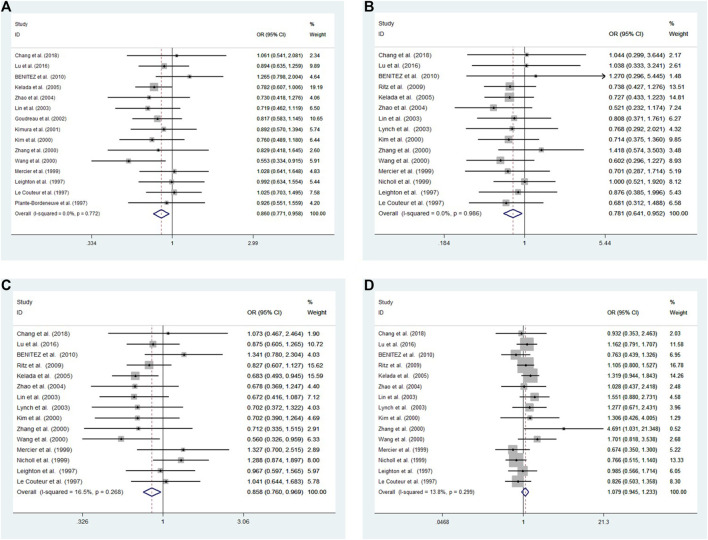
Meta-analysis with a fixed effects model for the association between the 3′-UTR VNTR in SLC6A3 and PD susceptibility. **(A)** Allele model, 10R vs. all others **(B)** Pseudodominant model, 10R/10R + 10R/9R vs. all others **(C)** Pseudorecessive model, 10R/10R vs. all others **(D)** Pseudocodominant model, 10R/9R vs. all others OR: odds ratio, CI: confidence interval, I-squared: measured to quantify the degree of heterogeneity in meta-analyses.

Cochran’s Q and I^2^ test results revealed low heterogeneity among studies in four models (10R vs. all others *p* = 0.772 I^2^ = 0.0%; 10R/10R + 10R/9R vs. all others: *p* = 0.986 I^2^ = 0.0%; 10R/10R vs. all others: *p* = 0.268 I^2^ = 16.5%; 10R/9R vs. all others *p* = 0.299 I^2^ = 13.8%, respectively) (Figure 2).

**FIGURE 3:**
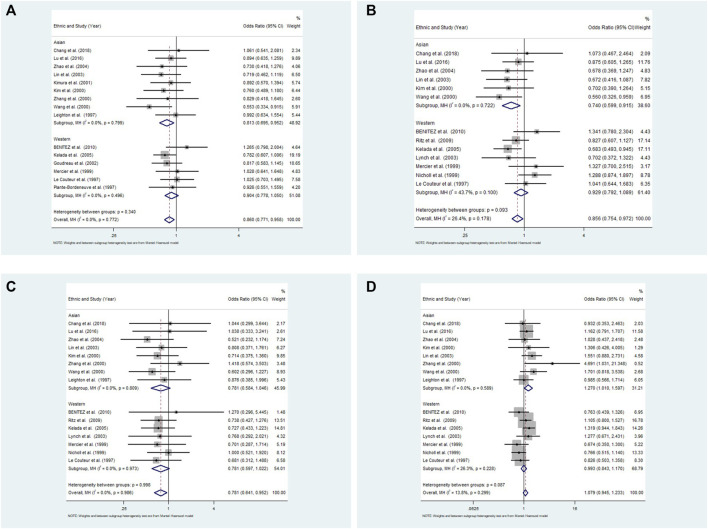
Meta-analysis with a fixed effects model for the association between the 3′-UTR VNTR in SLC6A3 and PD susceptibility in Asian and Western populations. **(A)** Allele model, 10R vs. all others; **(B)** Pseudodominant model, 10R/10R + 10R/9R vs. all others; **(C)** Pseudorecessive model, 10R/10R vs. all others; **(D)** Pseudocodominant model, 10R/9R vs. all others OR: odds ratio, CI: confidence interval, I-squared: measure to quantify the degree of heterogeneity in meta-analyses.

In the subgroup analysis by ethnicity, the results also revealed low heterogeneity among studies in four models in the Asian populations (10R vs. all others *p* = 0.799 I^2^ = 0.0%; 10R/10R + 10R/9R vs. all others: *p* = 0.809 I^2^ = 0.0%; 10R/10R vs. all others: *p* = 0.792 I^2^ = 0.0%; 10R/9R vs. all others *p* = 0.589 I^2^ = 0.0%, respectively) and in Western populations (10R vs. all others *p* = 0.496 I^2^ = 0.0%; 10R/10R + 10R/9R vs. all others: *p* = 0.973 I^2^ = 0.0%; 10R/10R vs. all others: *p* = 0.100 I^2^ = 43.7%; 10R/9R vs. all others *p* = 0.228 I^2^ = 26.3%, respectively) (Figure 3).

### The Association Between the 10-Repeat of the 3′-UTR VNTR in *SLC6A3* and PD

A fixed-effect model was used to analyze four models. The results showed a significant difference between patients and controls for the allele model (10R vs. all others: OR = 0.860, 95% CI: 0.771–0.958, *p* = 0.006), pseudodominant model (10R/10R + 10R/9R vs. all others: OR = 0.781, 95% CI: 0.641–0.952, *p* = 0.014) and pseudorecessive model (10R/10R vs. all others: OR = 0.858, 95% CI: 0.760–0.969, *p* = 0.013). No significant differences were observed under the pseudocodominant model (10R/9R vs. all others: OR = 1.079, 95% CI: 0.945–1.233, *p* = 0.262) (Figure 2).

In the subgroup analysis by ethnicity, the results showed a significant difference between patients and controls for the allele model (10R vs. all others: OR = 0.813, 95% CI: 0.695–0.952, *p* = 0.010), pseudorecessive model (10R/10R vs. all others: OR = 0.769, 95% CI: 0.637–0.928, *p* = 0.006) and pseudocodominant model (10R/9R vs. all others: OR = 1.270, 95% CI: 1.010–1.597, *p* = 0.041), but no significant differences were observed under the pseudodominant model (10R/10R + 10R/9R vs. all others: OR = 0.781, 95% CI: 0.584–1.046, *p* = 0.097) in Asian populations with a fixed-effect model. There was no significant difference between patients and controls for the four models in the Western populations **(**10R vs. all others: OR = 0.904, 95% CI: 0.778–1.050, *p* = 0.187; 10R/10R + 10R/9R vs. all others: OR = 0.781, 95% CI: 0.597–1.022, *p* = 0.071; 10R/10R vs. all others: OR = 0.929, 95% CI: 0.792–1.089, *p* = 0.361; 10R/9R vs. all others: OR = 0.993, 95% CI: 0.843–1.170, *p* = 0.930) (Figure 3).

### Publication Bias

**FIGURE 4:**
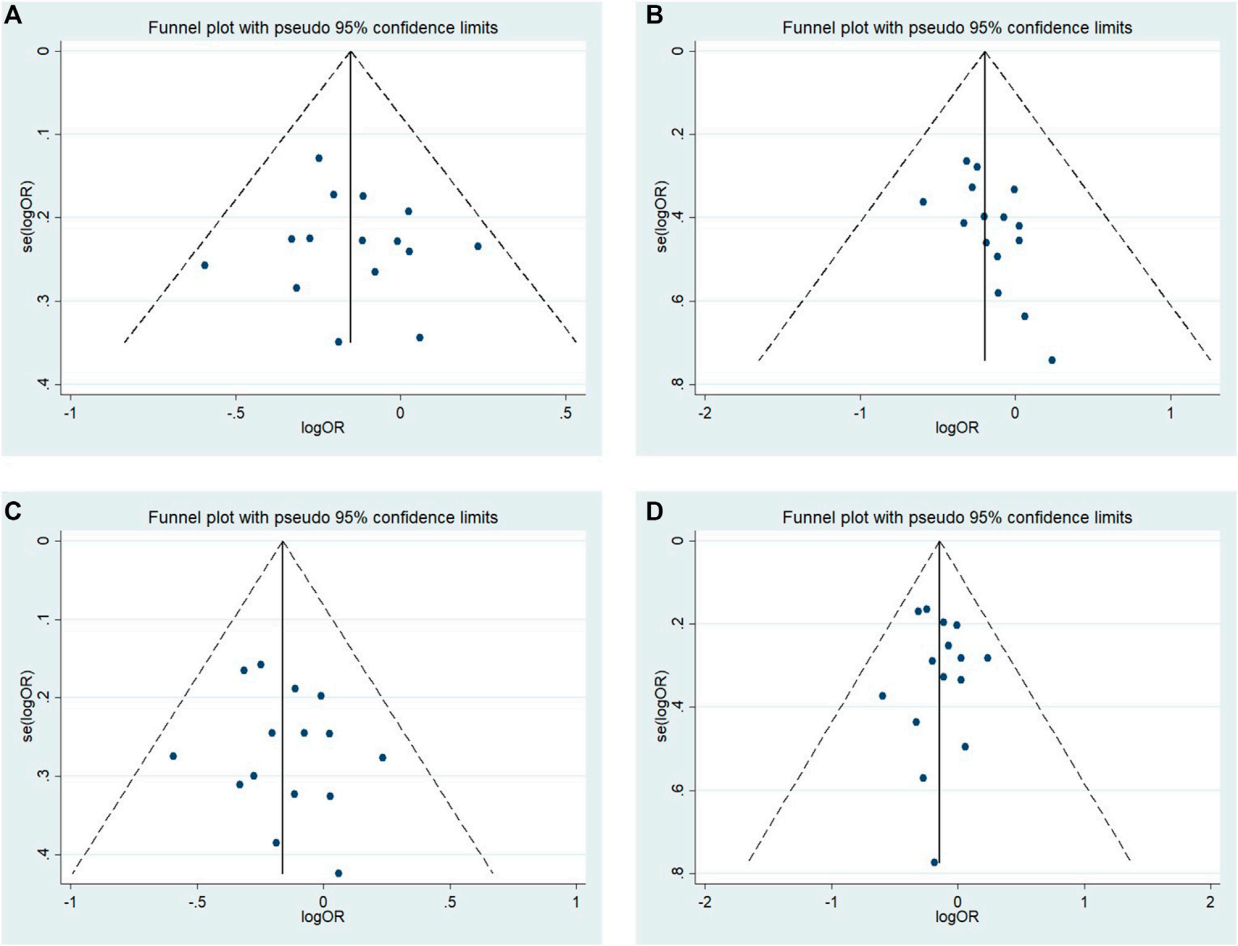
Funnel plot of the odds ratios in the meta-analysis. **(A)** Allele model, 10R vs. all others **(B)** Pseudodominant model, 10R/10R + 10R/9R vs. all others **(C)** Pseudorecessive model, 10R/10R vs. all others **(D)** Pseudocodominant model, 10R/9R vs. all others OR: odds ratio, CI: confidence interval, I-squared: measured to quantify the degree of heterogeneity in meta-analyses.

**FIGURE 5:**
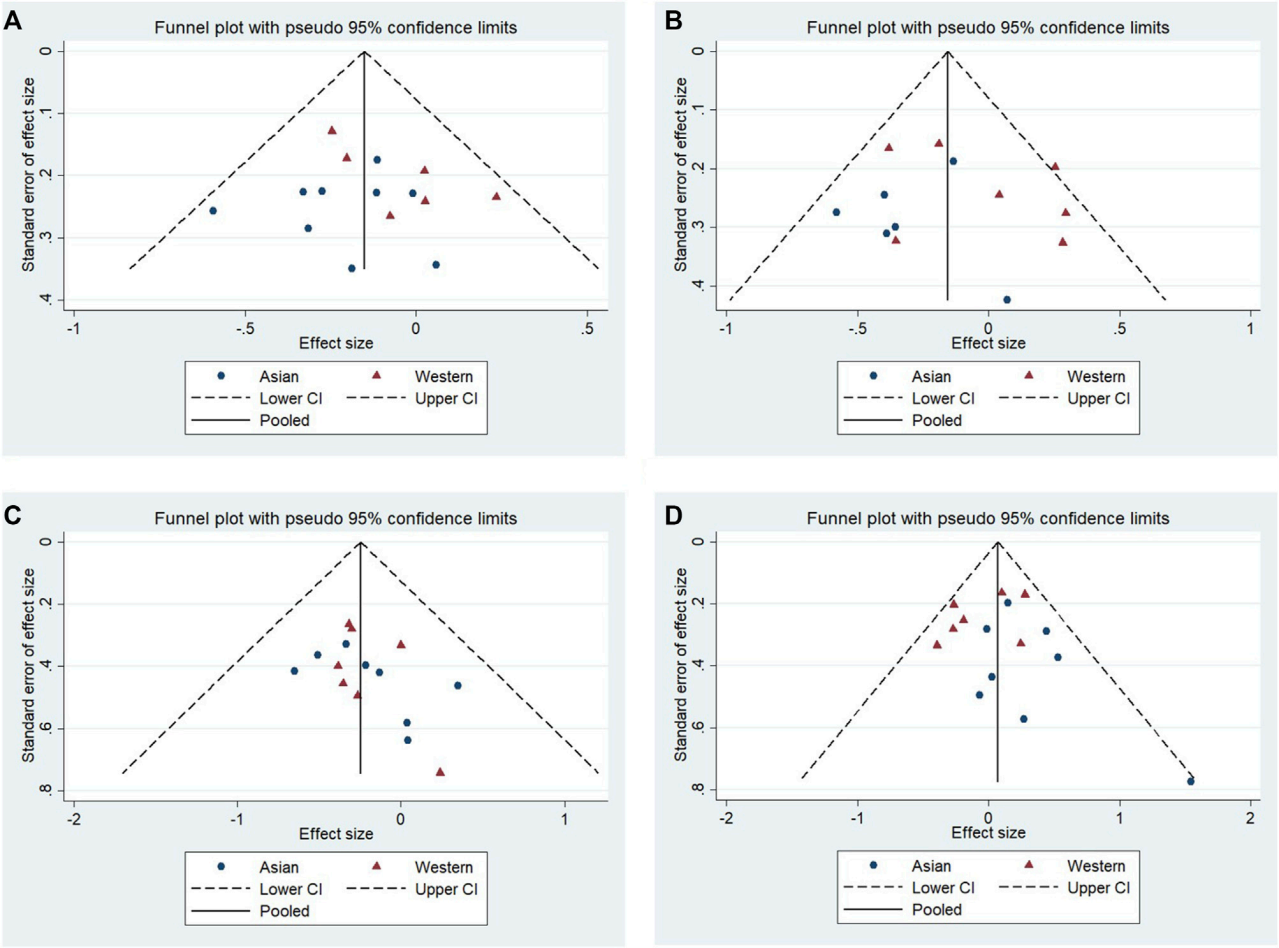
Funnel plot of the odds ratios in the subgroup: Asian populations and Western populations. **(A)** Allele model, 10R vs. all others. **(B)** Pseudodominant model, 10R/10R + 10R/9R vs. all others. **(C)** Pseudorecessive model, 10R/10R vs. all others. **(D)** Pseudocodominant model, 10R/9R vs. all others.

No significant publication bias was observed in any of the above genetic models via Begg’s funnel plot and Egger’s test (all *p* > 0.05, data not shown), and the funnel plot was symmetrical, with studies not coagulating into one quadrant of the funnel (Figures 4, 5).

## Discussion

This meta-analysis assessed the association between the 10R allele of the 3′-UTR VNTR in the *SLC6A3* gene and PD, and it included a total of 18 published studies. In general, our findings suggested that the 10R alleles and 10R/10R and 10R/10R + 10R/9R genotypes of the VNTR polymorphism in the *SLC6A3* gene confer protection against PD. The 10R alleles and 10R/10R genotype results were replicated in Asian populations, and the 10R/9R genotype was associated with an increased risk of PD in Asian populations. The current meta-analysis confirmed most of the previous findings showing that the 10R allele of the 3′-UTR VNTR in the *SLC6A3* gene may be a protective factor in susceptibility to PD.

Previous studies have shown that the prevalence of PD in Asia is low, approximately half that of Caucasians (Zhang and Román 1993; Leighton et al., 1997). This may be related to the discrepancies in genetic polymorphisms among populations of different racial and ethnic groups. There was a difference in allelic frequency in the *SLC6A3* VNTR polymorphism (Vandenbergh et al., 1992; Sano et al., 1993; Le Couteur et al., 1997; Leighton et al., 1997; Mercier et al., 1999; Kim et al., 2000) and the distribution was similar among the different Asian ethnic populations (Chinese, Korean and Japanese), but it was different from the Western populations. There are research findings that the frequencies of the 10 and 11 repeats of *SLC6A3* in Asian populations were higher than those in Caucasians, but the 9R of *SLC6A3* was much lower in normal Asian populations. The results of our meta-analysis indicate that the 10R may be a protective factor against susceptibility to PD in Asian populations, which may be one of the reasons for the low prevalence of PD in Asia.

Several studies indicate that the 9R allele demonstrates more enhanced transcription activity than the 10R allele of the *SLC6A3* VNTR polymorphism (Purcaro et al., 2019; Miller and Madras, 2002; Michelhaugh et al., 2001). From a clinical point of view, increased DAT expression due to the 9R allele might exacerbate striatal neuronal damage over time by increasing the presynaptic uptake of potentially neurotoxic endogenous or exogenous substrates via DAT, such as 1-methyl-4-phenyl-1,2,3,6-tetrahydropyridine (MPTP) (Contin et al., 2004; Tipton and Singer, 1993). However, the 10R/10R genotype of the *SLC6A3* gene may result in the most stable expression, which may confer nerve terminal protection against Mpp^+^-like compounds and prevent the toxicity of dopaminergic neurons (Lin et al., 2003). This may effectively reduce the incidence of PD. Moreover, interindividual genetic differences in DAT might also play a role in the therapeutic outcome of levodopa-treated PD patients (Contin et al., 2004). The DAT 9R allele has been suggested to be a predictor of dyskinesias or psychosis in PD patients (Kaiser et al., 2003). In general, research has shown that changes in the number of VNTR copies are closely related to PD, and our meta-analysis suggests that the 10R allele may be a protective factor in susceptibility to PD. We also conducted heterogeneity analysis, and we found low heterogeneity in our meta-analysis. In addition, our meta-analysis showed no publication bias.

There are potential limitations to the current study. First, PD is a complex disorder that develops as a result of age, environmental, and genetic factors, but age and exposure to environmental agents were often not discussed in our included studies. Moreover, interactions between multiple genes might affect the risk of PD. Additionally, since some are a bit ambiguous from the current Ethnic column (e.g., Ritz et al., Lynch et al.). Ritz’s study inclued 13 Asian populations, and Lynch‘s sudy inclued 9 other populations. Although these quantities account for a relatively small proportion of the total, these were difficult to conduct more accurate analyses. Therefore, the findings should be interpreted with caution. Further studies are necessary to establish larger sample sizes and consider SNP-SNP, gene–gene and gene–environmental interactions before reaching robust conclusions.

## Conclusion

Our findings suggest that the 10R of the 3′-UTR VNTR in *SLC6A3* may be a protective factor in susceptibility to PD. This result was also confirmed in Asian populations.

## Data Availability

The original contributions presented in the study are included in the article, further inquiries can be directed to the corresponding authors.
