# Corrigendum: The 10-Repeat 3′-UTR VNTR Polymorphism in the *SLC6A3* Gene May Confer Protection Against Parkinson’s Disease: A Meta-Analysis

**DOI:** 10.3389/fgene.2021.789112

**Published:** 2021-10-21

**Authors:** Qiaoli Zeng, Fan Ning, Shanshan Gu, Qiaodi Zeng, Riling Chen, Liuquan Peng, Dehua Zou, Guoda Ma, Yajun Wang

**Affiliations:** ^1^ Maternal and Children’s Health Research Institute, Shunde Women and Children’s Hospital, Guangdong Medical University, Foshan, China; ^2^ Key Laboratory of Research in Maternal and Child Medicine and Birth Defects, Guangdong Medical University, Foshan, China; ^3^ Institute of Neurology, Affiliated Hospital of Guangdong Medical University, Zhanjiang, China; ^4^ Department of Clinical Laboratory, People’s Hospital of Haiyuan County, Zhongwei, China; ^5^ Department of Pediatrics, Shunde Women and Children’s Hospital, Guangdong Medical University, Foshan, China; ^6^ Institute of Respiratory, Shunde Women and Children’s Hospital, Guangdong Medical University, Foshan, China

**Keywords:** Parkinson’s disease, Slc6a3, dopamine transporter, variable number of tandem repeats, metaanalysis

In the original article, there were some mistake in the **Legends** for FIGURE 2 | Meta-analysis with a fixed effects model for the association between the 3′-UTR VNTR in SLC6A3 and COPD susceptibility and FIGURE 3 | Meta-analysis with a fixed effects model for the association between the 3′-UTR VNTR in SLC6A3 and COPD susceptibility in Asian and Western populations as published. The “COPD” in the legends of Figures 2 and 3 should be “PD.” The correct legend appears below.

FIGURE 2 | Meta-analysis with a fixed effects model for the association between the 3′-UTR VNTR in SLC6A3 and PD susceptibility.

FIGURE 3 | Meta-analysis with a fixed effects model for the association between the 3′-UTR VNTR in SLC6A3 and PD susceptibility in Asian and Western populations.

Additionally, there were some minor formatting errors in **References**. Following references: Chang et al., 2018; Wang et al., 2000; Zhang et al., 2000; Zhao et al., 2004 as “(chinese),” should be “in Chinese” And the for reference: Lin et al., 2003, “Lin, J.-J., Yueh, K.-C., Chang, D.-C., Chang, C.-Y., Yeh, Y.-H., and Lin, S.-Z. (2003). The Homozygote 10-copy Genotype of Variable Number Tandem Repeat Dopamine Transporter Gene May Confer protection against Parkinson’s Disease “for Male, but “Not to Female Patients. J. Neurol. Sci. 209, 87–92. doi: 10.1016/s0022-510x(03)00002-9, it should be Lin, J. J., Yueh, K. C., Chang, D. C., Chang, C. Y., Yeh, Y. H., and Lin, S. Z. (2003). The homozygote 10-copy genotype of variable number tandem repeat dopamine transporter gene may confer protection against Parkinson’s disease for male, but not to female patients. J. Neurol. Sci. 209, 87–92. doi: 10.1016/s0022-510x(03)00002-9.”

Finally, **Figure 5** was incorrectly cited in the Discussion section. A correction has been made to Section: Discussion, Paragraph 1:

“This meta-analysis assessed the association between the 10R allele of the 3′-UTR VNTR in the SLC6A3 gene and PD, and it included a total of 18 published studies. In general, our findings suggested that the 10R alleles and 10R/10R and 10R/10R + 10R/9R genotypes of the VNTR polymorphism in theSLC6A3 gene confer protection against PD. The 10R alleles and 10R/10R genotype results were replicated in Asian populations, and the 10R/9R genotype was associated with an increased risk of PD in Asian populations. The current meta-analysis confirmed most of the previous findings showing that the 10R allele of the 3′-UTR VNTR in the SLC6A3 gene may be a protective factor in susceptibility to PD.”

The authors apologize for this error and state that this does not change the scientific conclusions of the article in any way. The original article has been updated.

